# Can a parental sleep intervention in an individual setting improve the maternal and paternal sense of competence and parent–child interaction in parents of young sleep-disturbed children? findings from a single-arm pilot intervention study

**DOI:** 10.1186/s40359-022-00945-y

**Published:** 2022-10-31

**Authors:** Marisa Schnatschmidt, Friederike Lollies, Angelika A. Schlarb

**Affiliations:** Faculty of Psychology and Sports Science, Department of Psychology, Clinical Psychology and Psychotherapy of Childhood and Adolescence, Bielefeld University, P.O.P. 10 01 31, DE-33501 Bielefeld, Germany

**Keywords:** Child’s sleep, Crying, Eating problems, Early regulatory problems, Parenting

## Abstract

**Background:**

In early childhood sleep and regulatory problems, parental factors are often impaired but essential to overcoming them. This study aims to examine, in parents of young sleep-disturbed children, whether mothers’ and fathers’ sense of parenting competence were increased and dysfunctional parent–child interactions reduced with a parental sleep intervention, whether these changes were sustained over a 12-month follow-up period and if children’s symptomatic parameters could be related factors.

**Methods:**

A total of 57 families with sleep-disturbed children aged 6 months to 4 years entered this single-arm pilot study. Each parent pair participated in six weekly individual face-to-face sessions of a multimodal cognitive-behavioral sleep intervention. The Parenting Sense of Competence Scale, Parental Stress Index Short Form, Child’s Sleep Diary and Child’s Questionnaire on Crying, Eating and Sleeping were obtained pre-, post-, 3, 6 and 12 months after the intervention.

**Results:**

Maternal sense of competence and dysfunctional mother–child interaction improved significantly up to 6 months after the intervention. Factors related to lower maternal competence were the child’s more frequent nightly food intake and more crying due to defiance; factors related to dysfunctional mother–child interaction were more frequent crying episodes, more crying due to defiance and more eating difficulties; factors related to increased maternal competence were less duration of child’s night waking, less bed-sharing and lower frequency of crying episodes; factors related to increased paternal competence were less child’s nightly food intake and fewer episodes of unexplained and unsoothable crying; and factors related to improved father–child interaction were less frequent child’s night waking and fewer unexplained and unsoothable crying episodes.

**Conclusion:**

For parents of sleep-disturbed young children, an intervention that addresses the child’s sleep could be promising to increase the parental sense of competence and reduce dysfunctional parent–child interactions, especially for mothers. Child symptomatic parameters may change, together with the parental sense of competence and parent–child interaction of both parents, after the intervention. Mothers with children with more severe symptomatology perceive their parenting competence as lower on average and their mother–child interaction as more dysfunctional. Future research with a larger sample and a randomized controlled design is needed.

**Trial registration:**

The study was retrospectively registered at the German Clinical Trials Register (ID: DRKS00028578; registration date: 21.03.2022).

## Background

Children’s sleep develops dynamically in the first years of life. By 6 months, infants usually develop a self-soothing ability that enables them to fall back to sleep on their own without help from their parents after the naturally occurring nighttime awakenings [1]. However, in the first years, 20–30% of children have problems falling and staying asleep [1]. Such early regulatory problems manifest mainly in problematic sleeping, crying and eating behavior and may also co-occur [2–6]. The prevalence of early regulatory problems varies in the range 10–63% [7–9].

As caregivers are directly involved in young children’s sleep and regulation problems, focusing on parental competence and parent–child interaction in interventions could be promising [1, 2, 6, 10−14].

Parental sense of competence includes parents’ perceptions of their ability in the parenting role (self-efficacy) and feelings and emotions associated with the parenting role (satisfaction) [15]. Previous research showed an association between a lower parental sense of competence and children’s sleep problems [14, 16−22] although others have not confirmed this [23, 24]. Furthermore, early regulatory problems are associated with a child’s difficult temperament and negative emotionality, which co-occur with lower parental competence [2, 25− 29].

Dysfunctional parent–child interactions often co-occur with early regulatory problems and are characterized by parental difficulties in handling the child’s regulatory problems that result in their maintenance or reinforcement and by parental dissatisfaction with their interactions [30, 31]. Lower parent–child interaction quality is associated with more child sleep problems [32–35]. However, other studies have found an inverse or no relationship [36–39]. Mothers and fathers could differ in the parental sense of competence and parent–child interaction [34, 40− 44].

The present study analyses the effects of a parent-focused intervention to improve children’s sleep that includes the following main components: parental knowledge about the child’s sleep; parenting skills training; relaxation exercises; and dysfunctional parental cognition reduction [45]. These elements are associated with improvements in children’s sleep that are bidirectionally related to the parental sense of competence and parent–child interaction [6, 11, 46, 47]. For example, parental knowledge about children’s sleep is associated with improved children’s sleep and an increased parental sense of competence [15, 46].

Early childhood sleep and regulation problems are associated with diverse short- and long-term consequences [19, 48−51]. Parenting factors are often impaired in early regulatory problems but play a key role in overcoming these transactional regulatory problems [4, 6, 11, 18, 46, 52, 53]. As problematic child behavior could predict future negative parenting practices, strengthening parental competencies is important [19, 54]. Additionally, there is a research gap regarding secondary outcomes and long-term assessment of pediatric sleep interventions [55]. Therefore, it is paramount to investigate maternal and paternal factors in treating early sleep and regulatory problems.

The purpose of this study is to examine, in parents of young children with sleep disturbances, whether mothers’ and fathers’ sense of parenting competence increase and dysfunctional parent–child interactions reduce with a parent-focused intervention to improve children’s sleep, whether these changes persist over the follow-up period of 12 months and if children’s symptomatic parameters are related to the sense of parenting competence and dysfunctional parent– child interactions over the follow-up period.

## Methods

### Participants and procedure

**Fig. 1:**
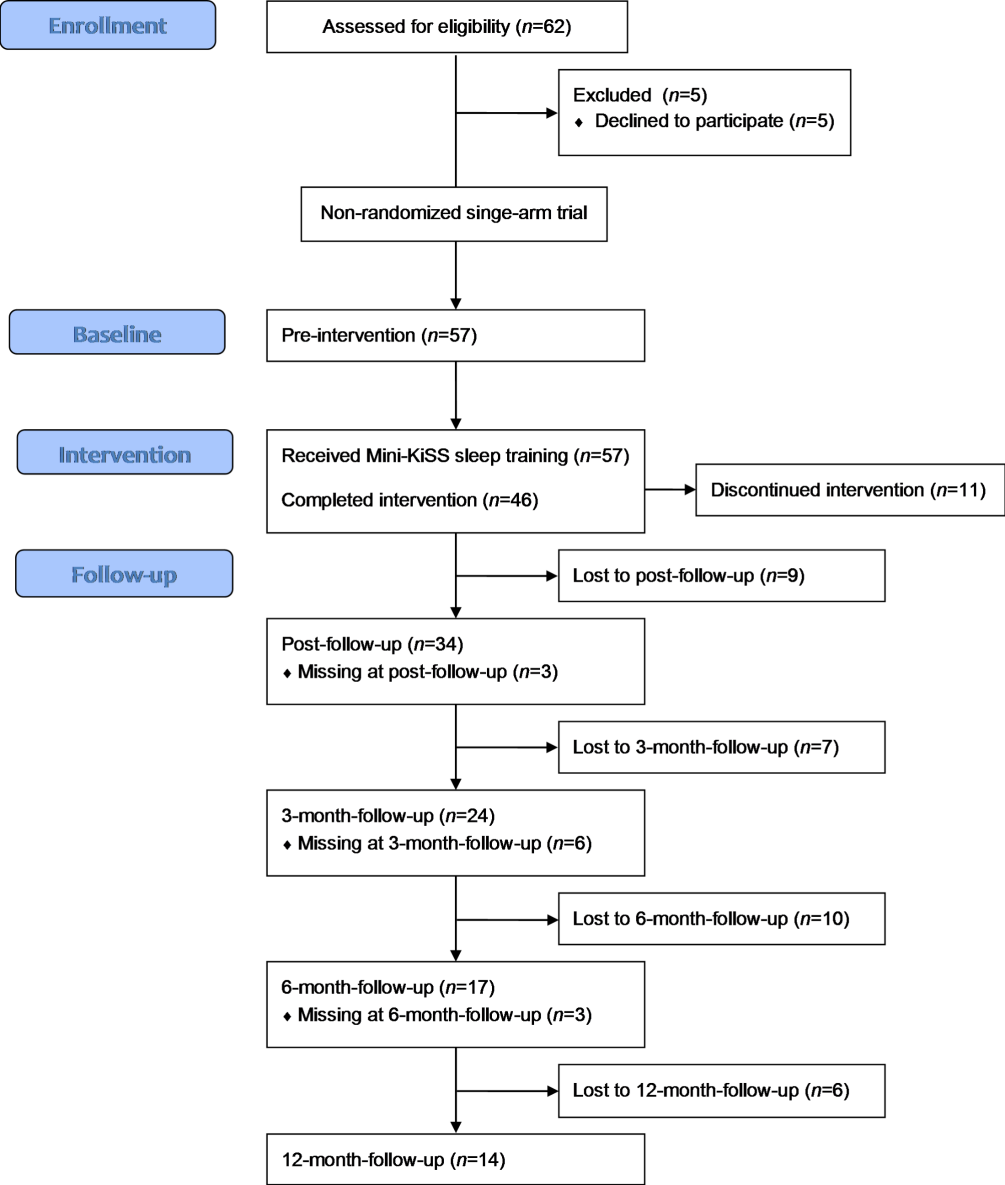
Flowchart of the families participating in the study, which include three families with two children and six families with single-parent mothers. A total of 62 families were screened, with five families deciding to participate at a later time point due to family reasons. “Missing data” refers to data from families who were missing at the corresponding measurement time point but were not completely dropped out of the study because their data were available at a later time point

Participants were 57 mothers and 51 fathers of 60 sleep-disturbed children (see Table 1 for sample characteristics). The recruiting was in the local area of Bielefeld, Germany, with the inclusion criteria displayed in Table 2. Participation was free of charge. The study design is illustrated in Fig. 1. The study was reviewed and approved by the Ethics Committee of Bielefeld University (No. 2017-036) and was in accordance with the Declaration of Helsinki. The study was retrospectively registered in the German Clinical Trials Register (ID: DRKS00028578; UTN: U1111-1275-7616).

**Table 1:** Sample characteristics

Sample characteristics	
Children (*N* = 60)	
Female child, *n* (%)	30 (50)
Age, months (Mdn Q1–Q3)	17 (12.00–26.75)
Mothers (*N* = 57)	
Age, years (mean, SD)	33.96 (4.42)
High-school or higher education (*n* = 56), *n* (%)	24 (42.9)
Employed (full- or part-time, *n* = 53), *n* (%)	31 (58.5)
Fathers (*N* = 51)	
Age, years (mean, SD)	36.00 (6.02)
High-school or higher education (*n* = 50), *n* (%)	25 (50.0)
Employed (full- or part-time, *n* = 47), *n* (%)	46 (94.6)

**Notes**: Mean and standard deviation are given when the data were normally distributed; if not, medians and interquartile ranges are provided. All values in percent refer to the available data. Three parent pairs had two children in the study and six mothers were single parents.**Abbreviations**: Mnd, median; Q1, 25th percentile; Q3, 75th percentile; SD, standard deviation

**Table 2:** Overview of the inclusion criteria

Inclusion criteria
• Children aged between 6 and 48 months
• Completed parental consent form
• Sleep disturbance diagnosis according to the International Classification of Sleep Disorders [56] and age-related criteria [57] o Sleep onset disturbance (≥ 2 episodes per week, with two of the three following criteria, over ≥ 4 weeks) ♣ Sleep onset latency over 30 min if the child was between 12 and 23 months or over 20 min if the child was older than 24 months ♣ Need for the parents in the room to fall asleep ♣ Bedtime resistance o Night waking disturbance (≥ 2 episodes per week, over ≥ 4 weeks) ♣ More than two nighttime awakenings if the child was between 12 and 23 months old or more than one if the child was older than 24 months ♣ More than 20 min awake during the night

### Intervention

The Mini-KiSS is a multimodal manual-based sleep training intervention for parents of young children with sleep disturbances based on age-appropriate cognitive-behavioral therapy.[45]. Trained psychologists conducted the six weekly sessions exclusively with one parent pair at a time in a face-to-face setting; this was the first time this setting was used because previous studies implemented online or face-to-face group training [47, 58, 59]. The intervention’s developer, Prof. Schlarb, supervised the sessions. The six sessions include: (1) psychoeducation on the child’s sleep; (2) parenting behavior and sleep hygiene; (3) child crying and defiant behaviors; (4) parent and child stress and relaxation; (5) feelings of anxiety and security; and (6) relapse risk and summary^45^. The Mini-KiSS sleep training might be effective on children’s sleep and sleep-related parenting strategies [47, 58−60].

### Instruments

An overview of the instruments, scales and item examples is provided in Table 3.

**Table 3:** Overview of the instruments and scales used in the present study, with descriptions of the assessed construct and item examples

Instrument / Scale	Construct	Example items
Parental sense of competence [15]		
- Total score	Parental sense of competence, including self-efficiency and satisfaction with the parental role	• A difficult problem in being a parent is not knowing whether you are doing a good or a bad job • Being a parent is manageable and any problems are easily solved
Parental Stress Index Short Form [30]		
- Parent–child dysfunctional interaction subscale	Parental dissatisfaction with their interactions with their children	• My child does not like or want to be close to me • My child smiles at me less than expected
Sleep diary [59]		
- Sleep onset latency (SOL) - Frequency and duration of nighttime awakenings (FNW; DNW) - Bed-sharing (BS) - Nighttime food intake (NFI)	Children’s sleep behavior	• When did your child wake up at night and how long was he or she awake? • How long did it take for your child to fall asleep?
Questionnaire of Crying, Sleeping and Feeding [62]		
- Frequency of crying episodes (FRQ)	Frequency of crying episodes over the day	• How often does your child cry and whine for half an hour or more and cannot be soothed [one time of the day per item]?
- Unexplained and unsoothable crying (UUC)	Frequency of unexplained and unsoothable crying episodes	• How often do you feel that you can identify the cause of your child’s crying? • How often does your child respond to the calming support you provide?
- Crying due to defiance (DEF)	Frequency of crying episodes due to defiance	• If your child cries persistently and is difficult to calm, how often do you have the impression that the cause could be defiance?
- Feeding/eating subscale (EAT)	Difficulties in the eating situations with the child in general, the parental burden and whether there are any concerns about the weight of the child	• It takes my child more than 45 min to eat • My child eats only when distracted (playing, watching TV, listening to music)

#### Parenting sense of competence scale

The parenting sense of competence was evaluated with the German version of the Parenting Sense of Competence Scale [15, 61]. The questionnaire includes 16 items focusing on the parental perception of their competence in rearing their child. Mothers and fathers filled out the questionnaire independently, giving answers on a six-point Likert scale from 1 (strongly disagree) to 6 (strongly agree). In the present study, the total score is of interest, with high values indicating high parental perceived competence. The range of normative values is 59–77 for mothers and 65–83 for fathers [61]. Miller postulated good internal consistency of the total score (α = 0.76) [61], which is in line with the internal consistency in the present sample (mothers: α = 0.78–0.82; fathers: α = 0.71–0.78).

#### Parental stress index short form

The parent–child interaction was rated by mothers and fathers separately at one subscale of the German version of the well-established Parental Stress Index Short Form [30]. The parent–child dysfunctional interaction subscale includes 12 items and focuses on parental perception of their interactions with their child. The answers were given on a five-point scale from “strongly agree” to “do not agree at all”. A high score indicates more difficulties in parent–child interaction and the normal range is between 15 and 33 [30]. The internal consistency of this subscale is postulated as good (α = 0.80) [30], which is reflected in the present sample (mothers: α = 0.73–0.82; fathers: α = 0.65–0.87).

#### Children’s Sleep Diary

Children’s sleep was assessed with a sleep diary completed by parents [59]. Parents reported their child’s sleep over 14 days at each measurement time point. Usually, the second week was analyzed when there were no special events, such as illness, to ensure that the documentation was less biased and that a typical week was analyzed. The variables of interest are shown in Table 3.

#### Children’s questionnaire on crying, feeding and sleeping

With the German questionnaire on crying, feeding and sleeping [62], the child’s crying and eating problems were evaluated. The parents answered questions on children’s crying and eating during a typical week on a four-point scale (1, never/seldom; 4, always/daily), with high values indicating more difficulties. We referred to seven items concerning crying as the following factors [60]: frequency of crying episodes (FRQ: four items), unexplained and unsoothable crying (UUC: two items) and crying out of defiance (DEF: one item, including early defiance, which helps the child to regulate tension and is not intentional, and later intentional defiance [63]). Problematic eating behavior was assessed with the feeding subscale (EAT: 13 items). Normative values for the studied age group are not yet available. The internal consistencies of these scales in the present sample could be rated as mainly acceptable to good (FRQ: *α* = 0.72–0.85; UUC: *α* = 0.53–0.84; EAT: *α* = 0.72–0.87).

### Statistical analyses

All analyses were calculated with the Statistical Package for the Social Sciences SPSS 27.0 for Windows [64]. First, we analyzed differences in the outcome variables at baseline regarding the child’s age, child’s gender, attrition and between mothers and fathers due partly to non-normal distributed data and small sample size with the Kruskal-Wallis, Mann-Whiney U and Wilcoxon signed-rank tests. Furthermore, Wilcoxon signed-rank tests were calculated to examine the difference between the baseline, post- and follow-up measurements. The *p* values were adjusted according to Benjamini-Hochberg.

Linear mixed models (LMMs) were calculated to evaluate between-subject and within-subject factors. We chose LMMs because they are appropriate for the nested data structures and their robustness to missing data and violations of distribution assumptions [65]. Four LMMs were calculated separately for the maternal and paternal sense of competence and parent–child interaction. The fixed effects in each model were the children’s sleep, crying and eating parameters, each included as both between-subject and within-subject factors. Each model controlled for the covariates of time, children’s initial age and children’s gender. All models were specified with two levels (Level 2: measurement points; Level 1: individuals), a random intercept, the maximum likelihood estimation procedure and the forward-stepping strategy [66] We performed the available case analyses [67] and these analyses were calculated without *p* value adjustment because of the pilot nature of the study. Effect sizes are presented as Pearson’s *r* and Cohen’s *d* (with *r* = 0.1, 0.3 and 0.5 and *d* = 0.2, 0.5 and 0.8, describing small, medium and large effects).

## Results

As shown in Fig. 1, of the 57 families, 20 participated in the premeasurement only, while 37 families completed at least one additional postintervention measurement. The outcome variables at baseline do not differ significantly between study dropouts and non-dropouts or according to the age and gender of the child (Table 4). At baseline, there was no difference between mothers and fathers in their parental sense of competence (*z* = 1.76; *p* = 0.078), but mothers had more dysfunctional parent–child interactions (*z* = 2.19; *p* = 0.028) than fathers. Descriptive statistics of the relevant variables across the five measurement points are shown in Table 5.

**Table 4:** Descriptive statistics for outcomes at baseline across children’s age groups, gender and sample attrition

	Children’s age at baseline (T1) in months								Children’s gender				Attrition			
	6–11		12–23		24–35		36–48		Female		Male		Dropout		Non-dropout	
	*n*	Mdn (Q1–Q3)	*n*	Mdn (Q1–Q3)	*n*	Mdn (Q1–Q3)	*n*	Mdn (Q1–Q3)	*n*	Mdn (Q1–Q3)	*n*	Mdn (Q1–Q3)	*n*	Mdn (Q1–Q3)	*n*	Mdn (Q1–Q3)
Parental sense of competence																
Mothers	16	76.00 (65.75–79.75)	22	66.50 (61.50–75.75)	10	63.50 (60.25–75.75)	4	67.88 (53.50–70.44)	24	68.38 (64.25–73.25)	29	68.00 (60.50–79.50)	20	70.00 (62.75–77.25)	36	67.50 (61.25–78.00)
Fathers	15	74.67 (66.00–84.00)	19	69.00 (64.00–78.00)	9	78.00 (70.00–82.00)	4	76.50 (60.25–78.50)	22	67.50 (64.00–77.25)	26	76.00 (66.00–80.00)	18	75.00 (67.75–81.75)	33	73.00 (64.00–78.50)
Dysfunctional parent–child interaction																
Mothers	16	15.00 (13.25–18.00)	22	16.68 (14.00–19.25)	10	17.00 (16.00–21.75)	5	20.00 (16.00–31.00)	25	17.00 (14.00–18.50)	29	16.36 (15.00–20.00)	20	16.00 (14.25–18.00)	37	18.00 (14.00–20.50)
Fathers	14	15.00 (13.75–18.25)	19	15.00 (14.00–17.00)	9	16.00 (14.00–18.00)	4	14.50 (12.50–24.75)	22	15.00 (14.00–19.75)	25	15.00 (14.00–16.50)	18	15.00 (14.00–16.25)	32	15.00 (14.00–18.75)

**Notes**: “Dropout” refers to participants who dropped out after T1. “Non-dropout” refers to participants who completed at least T1 and one additional measurement point. Due to the small sample size and partly non-normal distribution, the outcomes were tests with Kruskal-Wallis (children’s age) and Mann-Whitney U tests. For the differences between children’s age and gender, only parents with one child in the study were analyzed. Therefore, three parent pairs were excluded from these analyses because they had two siblings in the study. There were no significant differences in the outcome variables (*p* > 0.05). Normative values are: for maternal sense of competence, 59–77; for paternal sense of competence, 65–83; for parent–child dysfunctional interaction, 15–33. **Abbreviations**: Mdn, median; Q1, 25th percentile; Q3, 75th percentile

**Table 5:** Descriptive statistics for the outcome and predictor variables at all measurement points

	T1		T2		T3		T4		T5	
	*N*	Mdn (Q1–Q3)	*n*	Mdn (Q1–Q3)	*n*	Mdn (Q1–Q3)	*n*	Mdn (Q1–Q3)	*n*	Mdn (Q1–Q3)
Parental sense of competence										
Mothers	56	68.38 (62.00–78.00)	33	77.00 (68.00–81.50)	23	78.00 (66.00–87.00)	16	74.50 (69.00–81.75	13	72.00 (61.50–81.50)
Fathers	51	74.00 (66.00–79.33)	27	76.00 (67.00–82.00)	19	73.00 (66.00–78.00)	14	74.00 (65.75–79.25)	11	75.00 (63.00–82.00)
Parent–child dysfunctional interaction										
Mothers	57	17.00 (14.00–20.00)	33	16.00 (13.50–18.50)	23	16.36 (14.00–20.00)	16	16.50 (13.25–18.75)	13	15.27 (13.50–20.00)
Fathers	50	15.00 (14.00–18.00)	28	15.00 (12.25–17.75)	20	14.00 (13.00–16.75)	14	13.04 (12.75–15.25)	11	15.00 (13.00–19.00)
Children’s sleeping										
SOL	59	26.43 (16.43–38.57)	36	19.28 (7.86–37.68)	25	20.00 (7.50–28.93)	18	18.00 (5.32–33.74)	14	27.14 (12.18–43.57)
FNW	59	2.29 (1.29–4.00)	36	1.07 (0.43–2.53)	25	1.14 (0.14–2.23)	18	0.50 (0.00–1.80)	14	1.00 (0.39–2.03)
DNW	59	26.43 (11.67–51.43)	36	11.93 (2.32–21.39)	25	5.71 (0.92–26.43)	18	3.33 (0.00–18.92)	13	9.29 (3.21–16.43)
BS	58	0.57 (0.14–1.00)	36	0.07 (0.00–0.66)	25	0.00 (0.00–0.29)	18	0.07 (0.00–1.00)	14	0.50 (0.10–0.60)
NFI	58	0.86 (0.43–1.00)	36	0.21 (0.00–0.86)	25	0.14 (0.00–0.91)	18	0.00 (0.00–0.57)	14	0.36 (0.10–0.60)
Children’s crying										
FRQ	60	1.50 (1.06–2.25)	35	1.25 (1.00–2.00)	24	1.00 (1.00–1.50)	19	1.00 (1.00–1.75)	14	1.50 (1.00–2.06)
UUC	60	2.00 (2.00–3.00)	35	2.00 (1.50–2.50)	24	1.50 (1.00–2.50)	18	1.75 (1.37–2.62)	14	2.50 (2.00–3.25)
DEF	58	2.00 (2.00–3.00)	35	2.00 (2.00–3.00)	24	2.00 (1.25–3.00)	19	2.00 (2.00–3.00)	14	2.50 (2.00–3.00)
Children’s eating										
EAT	60	1.48 (1.23–1.77)	33	1.31 (1.15–1.73)	24	1.39 (1.23–1.59)	17	1.15 (1.07–1.54)	14	1.27 (1.14–1.46)

**Notes**: Medians and quartiles of the parental sense of competence and parent–child dysfunctional interaction for mothers and fathers and children’s symptom characteristics (sleep, crying and eating patterns) through all five measurement points. Three parent pairs had two children in the study and six mothers were single parents. Normative values are: for maternal sense of competence, 59–77; for paternal sense of competence, 65–83; for parent–child dysfunctional interaction, 15–33. SOL and DNW are displayed in minutes; FNW in numbers per night. The critical values of SOL, DNW and FNW are shown in Table 2. BS and NFI represent the mean of nights in a week when BS or NFI did (1) or did not (0) occur. For the FRQ, UUC, DEF and EAT scales, normative values were not yet available for this age group. A value of 1 indicates that crying/eating problems occur seldom to never, a value of 2 indicates an occurrence of 1–3 times a week and a value of 3 indicates an occurrence of 4–6 times a week.**Abbreviations**: Mnd, median; Q1, 25th percentile; Q3, 75th percentile; T1, pre-intervention; T2, post-intervention; T3, 3-month follow-up; T4, 6-month follow-up; T5, 12-month follow-up; SOL, sleep onset latency; FNW, frequency of nightly awakening; DNW, duration of nightly awakening; BS, bed-sharing; NFI, nightly food intake; FRQ, crying frequency; UUC, unexplained and unsoothable crying; DEF, crying due to defiance; EAT, difficulties in eating behavior

### Parental parameters after the intervention

Maternal sense of competence increased significantly shortly after the sleep intervention, which remained significant over the follow-up period up to 6 months (Table 6). Mothers’ dysfunctional parent–child interaction decreased significantly until 3 months after the sleep intervention (Table 6). Among fathers, no significant change in their sense of competence or their father–child interaction was found (Tables 5 and 6).

**Table 6:** Results of Wilcoxon signed-rank tests for pre-intervention (T1) and post-intervention (T2–T5) differences

	Mothers				Fathers			
	*n*	*z*	*p*	*r*	*n*	*z*	*p*	*r*
Parental sense of competence								
T1–T2	32	3.69	0.000	0.65	27	1.97	0.091	0.38
T1–T3	23	3.64	0.000	0.76	19	0.26	0.430	0.06
T1–T4	16	2.64	0.008	0.66	14	0.18	0.430	0.05
T1–T5	13	1.47	0.095	0.41	11	0.18	0.430	0.05
Parent–child dysfunctional interaction								
T1–T2	33	−3.54	0.000	0.62	27	−1.11	0.267	0.21
T1–T3	23	−2.02	0.034	0.42	19	−1.86	0.091	0.43
T1–T4	16	−1.37	0.098	0.34	14	−1.83	0.091	0.49
T1–T5	13	−1.19	0.118	0.33	11	−0.36	0.430	0.11

**Notes**: The pre-, post- and follow-up analyses of maternal and paternal sense of competence and parent–child dysfunctional interaction. Wilcoxon signed-rank tests were calculated; *p* values are one-tailed due to the directional hypotheses and Benjamini-Hochberg adjusted. **Abbreviations**: T1, pre-intervention; T2, post-intervention; T3, 3-month follow-up; T4, 6-month follow-up; T5, 12-month follow-up

### Child’s symptom parameters as related factors

The intraclass correlation coefficients calculated from the intercept-only models indicated that 52.0–69.3% of the variance of the outcome variables was explained by the differences between the individuals. The final models of the LMM analyses are presented in Tables 7 and 8.

**Table 7:** Final model estimates of main effects for maternal and paternal sense of competence

	Mothers			Fathers		
	β (95% CI)	*t* (df)	*d*	β (95% CI)	*t* (df)	*d*
Between-subject factors (child)						
Sleep onset latency (SOL)						
Frequency of nightly awakening (FNW)						
Duration of nightly awakening (DNW)						
Bed-sharing (BS)						
Nightly food intake (NFI)	−8.858 (−13.972, −3.744)	−3.74 (49.89)***	0.99			
Frequency of crying episodes (FRQ)						
Unexplained and unsoothable crying (UUC)						
Crying due to defiance (DEF)	−3.067 (−6.126, −0.008)	−2.01 (58.19)*	0.53			
Eating difficulties (EAT)						
Within-subject factors (child)						
Sleep onset latency (SOL)						
Frequency of nightly awakening (FNW)						
Duration of nightly awakening (DNW)	−0.093 (−0.150, −0.035)	−3.22 (69.51)**	0.77			
Bed-sharing (BS)	−3.673 (−7.15, −0.195)	−2.11 (68.45)*	0.99			
Nightly food intake (NFI)				−2.585 (−4.923, −0.247)	−2.25 (32.54)*	0.79
Frequency of crying episodes (FRQ)	−2.904 (−5.060, −0.748)	−2.68 (75.73)**	0.62			
Unexplained and unsoothable crying (UUC)				−1.755(−3.039, −0.471)	−2.74 (50.26) **	0.77
Crying due to defiance (DEF)						
Eating difficulties (EAT)						
Covariates						
Age at baseline (child)	−0.232 (−0.447, −0.016)	−2.16 (50.47)*	0.61			
Gender (child)						
Time						

**Notes**: Due to the pilot nature of the study, no *p* value adjustment was applied: * *p* < 0.05; ** *p* < 0.01; *** *p* < 0.001

**Table 8:** Final model estimates of main effects for maternal and paternal parent–child dysfunctional interaction

	Mothers			Fathers		
	β (95% CI)	*t* (df)	*d*	β (95% CI)	*t* (df)	*d*
Between-subject factors (child)						
Sleep onset latency (SOL)						
Frequency of nightly awakening (FNW)						
Duration of nightly awakening (DNW)						
Bed-sharing (BS)						
Nightly food intake (NFI)						
Frequency of crying episodes (FRQ)	3.189 (1.012–5.360)	2.93 (62.77)**	0.99			
Unexplained and unsoothable crying (UUC)						
Crying due to defiance (DEF)	2.860 (1.398−4.322)	3.91 (61.40)***	0.75			
Eating difficulties (EAT)	2.875 (0.025−5.725)	2.02 (52.38)*	0.56			
Within-subject factors (child)						
Sleep onset latency (SOL)						
Frequency of nightly awakening (FNW)				0.688 (0.298−1.079)	3.51 (91.11)***	0.73
Duration of nightly awakening (DNW)						
Bed-sharing (BS)						
Nightly food intake (NFI)						
Frequency of crying episodes (FRQ)						
Unexplained and unsoothable crying (UUC)				0.430 (0.025−0.836)	2.16 (32.33)*	0.76
Crying due to defiance (DEF)						
Eating difficulties (EAT)						
Covariates						
Age at baseline (child)						
Gender (child)						
Time						

**Notes**: Due to the pilot nature of the study, no *p* value adjustment was applied: * *p* < 0.05; ** *p* < 0.01; *** *p* < 0.001

Mothers with children who were fed more often at night, who cried more due to defiance and who were older on average had lower scores on the parental sense of competence scale (Table 7). When the children were awake for a shorter time at night, slept less in the parent’s bed and cried less frequently, the mothers showed a higher parental sense of competence (Table 7). There were no significant between-subject factors for the paternal sense of competence among fathers. However, fathers showed higher scores in their parental sense of competence when children had less food during nighttime and cried less unexpectedly and unsoothably (Table 7).

Mothers with children who cried more often and more out of defiance and whose children had more eating difficulties on average rated their parent–child interaction as more dysfunctional (Table 8). However, fathers rated their parent–child interactions as more dysfunctional when children woke up more frequently and showed more unexplained and unsoothable crying (Table 8). No within-subject factors for mothers and no between-subject factors for fathers were significant.

## Discussion

This study aims to examine, in parents of young sleep-disturbed children, whether mothers’ and fathers’ sense of parenting competence were increased and dysfunctional parent–child interactions reduced in the short- and long-term after a parental intervention for child’s sleep and if the child’s symptomatic parameters were related factors.

### Effectiveness of the intervention

The maternal sense of parenting competence was significantly improved compared to baseline up to 6 months after the intervention, which is consistent with previous studies [47 ,68−71]. It is conceivable that the intervention improved parental knowledge about children’s sleep and their parenting skills, which may have contributed to the increased maternal sense of competence. However, the present study did not identify any improvement in the paternal sense of competence, which contrasts with previous research [47, 71] The comparability of these studies may be limited and it is possible that the fathers in the sample studied were less involved in daily child care and thus experienced fewer situations in which they could experience a change in their parenting skills.

The dysfunctionality of parent–child interactions in mothers was reduced up to 3 months after the intervention, which follows previous studies [72–74]. It could be hypothesized that this change was due to both a reduction in children’s sleep and regulation problems and improved parental coping with these problems, which may have benefited from key elements of the intervention, such as parental skills training, relaxation exercises and a reduction in dysfunctional parental cognitions. In contrast, father–child interaction did not improve. This finding was surprising because fathers were shown to improve their parenting coping strategies after a similar intervention [47, 75]. As fathers rated their parent–child interaction as being less dysfunctional at baseline than mothers, an improvement may have been less likely due to floor effects.

### Related factors for the parental sense of competence

The study findings suggest that children’s sleep and crying parameters changed, together with the parental sense of competence of both parents, after the intervention. In addition, the results indicate that mothers with children who had more problematic sleep and crying behaviors on average had a lower sense of parental competence. Possible explanations could be that the significant child’s sleep factors are related to disruptions in parental sleep, which are related to a lower parental sense of competence [76–79]. Furthermore, parents may aim to reduce night feeding and feel that it strains their parenting competence if they cannot soothe the child with alternative strategies [23, 45, 53, 80]. Also, crying out of defiance is associated with increased parental stress, which is related to a lower parental sense of competence [63, 81, 82]. Furthermore, infants’ and toddlers’ negative emotionality were longitudinally linked to lower self-efficiency in mothers’ and fathers’ parenting [83].

### Related factors for parent–child interaction

The child’s sleep and crying behavior improved together with the father–child interaction and more problematic child’s crying and eating patterns on average co-occur with more dysfunctional mother–child interactions. These findings align with previous research [34,84–86]. One explanation could be that situations with crying and difficult mealtimes could be stressful for parents, which is associated with dysfunctional parent–child interactions [87–90]. Furthermore, the results contribute to the hypotheses that positive parent–child interaction is essential for child self-regulation, which affects sleep behavior, and that fathers play an important role in child sleep behavior. However, in the present study, child sleep was not related to mother–child interaction, which is in contrast to Bordeleau and colleagues but aligns with other studies [34, 38, 39]. It is possible that in the analyzed sample the mothers took on the main tasks of caregiving and thus can include more diverse parent–child interactions, whereas the fathers, due to full employment, may spend less time with the child, which tends to occur in the evening, making the connection with the child’s sleep more plausible.

However, the relation between children’s regulatory problems, parental competence and parent–child interaction could be bidirectional [6, 11, 52, 91−93].

## Limitations

This study addresses the research gap of longitudinal secondary outcomes of both parents following a parental intervention to improve young children’s sleep. However, because of several limitations, the findings should be interpreted with caution. First, the analyses include a small sample characterized by a substantial attrition rate; a detailed analysis of the sample attrition with recommendations for future studies can be found elsewhere^60^. Second, the study design did not include any comparison to a control group, therefore the study findings cannot be interpreted as direct effects of the intervention. Other possible effects, such as the developmental transience of problems, could also cause the present findings [94, 95]. Third, the sample includes a wide child age range to avoid having small subsamples, which could complicate the interpretation of the study results due to the different developmental stages [1, 6]. Fourth, the data were based on questionnaires and diaries completed by the parents, which could bias the data. In addition, we did not record whether the child’s data were completed by the mother or the father. Furthermore, the results of the LMM analyses may have an increased risk of Type I error because they were performed without *p* value adjustment. Future studies should implement more objective data, such as actigraphy for sleep parameters or videotaped sequences to analyze children’s regulatory patterns and parental behavior [96]. However, this pilot study provides a basis for implementing a randomized clinical trial with a larger sample size to verify and extend the present findings.

## Conclusion

A parent-focused intervention to improve young child’s sleep problems could be a promising approach to increase the parental sense of competence and reduce dysfunctional parent–child interactions, especially for mothers. Children’s symptoms may change together with the parental sense of competence and parent–child interaction. Mothers of children with more severe symptomatology on average may perceive their parenting competence as lower and their mother–child interaction as more dysfunctional. These results are relevant for clinical practice because the treatment of early childhood sleep and regulatory problems often needs to address a disturbed parental sense of competence and dysfunctional parent–child interactions, which can be well implemented with a manualized therapy program. Future randomized controlled trials with larger samples are needed to strengthen the present findings.

## Data Availability

The datasets generated and analyzed during the current study are available from the corresponding author on reasonable request.

## List of abbreviations

BS: bed-sharing.
DEF: crying out of defiance.
DNW: duration of nighttime awakenings.
EAT: difficulties in eating behavior.
FNW: frequency of nighttime awakenings.
FRQ: frequency of crying episodes.
LMMs: linear mixed models.
NFI: nighttime food intake.
SFS: Fragebogen zum Schreien, Füttern, Schlafen (questionnaire on crying, feeding and sleeping).
SOL: sleep onset latency.
T1: measurement point pre-intervention.
T2: measurement point post-intervention.
T3: measurement point 3 months after the intervention.
T4: measurement point 6 months after the intervention.
T5: measurement point 12 months after the intervention.
UUC: unexplained and unsoothable crying.
